# Supplementary material for: Comprehensive Annotation and Functional Exploration of MicroRNAs in Lettuce
Source: Front Plant Sci. 2021 Dec 24;12:781836. doi: 10.3389/fpls.2021.781836 (PMC8739914; doi:10.3389/fpls.2021.781836)
Supplement: Supplementary file 2 [file Data_Sheet_8.ZIP › GSM2230749.plot/Lsa-miR172a_Lsat_1_v5_gn_8_18040.1_1210_TPlot.pdf]

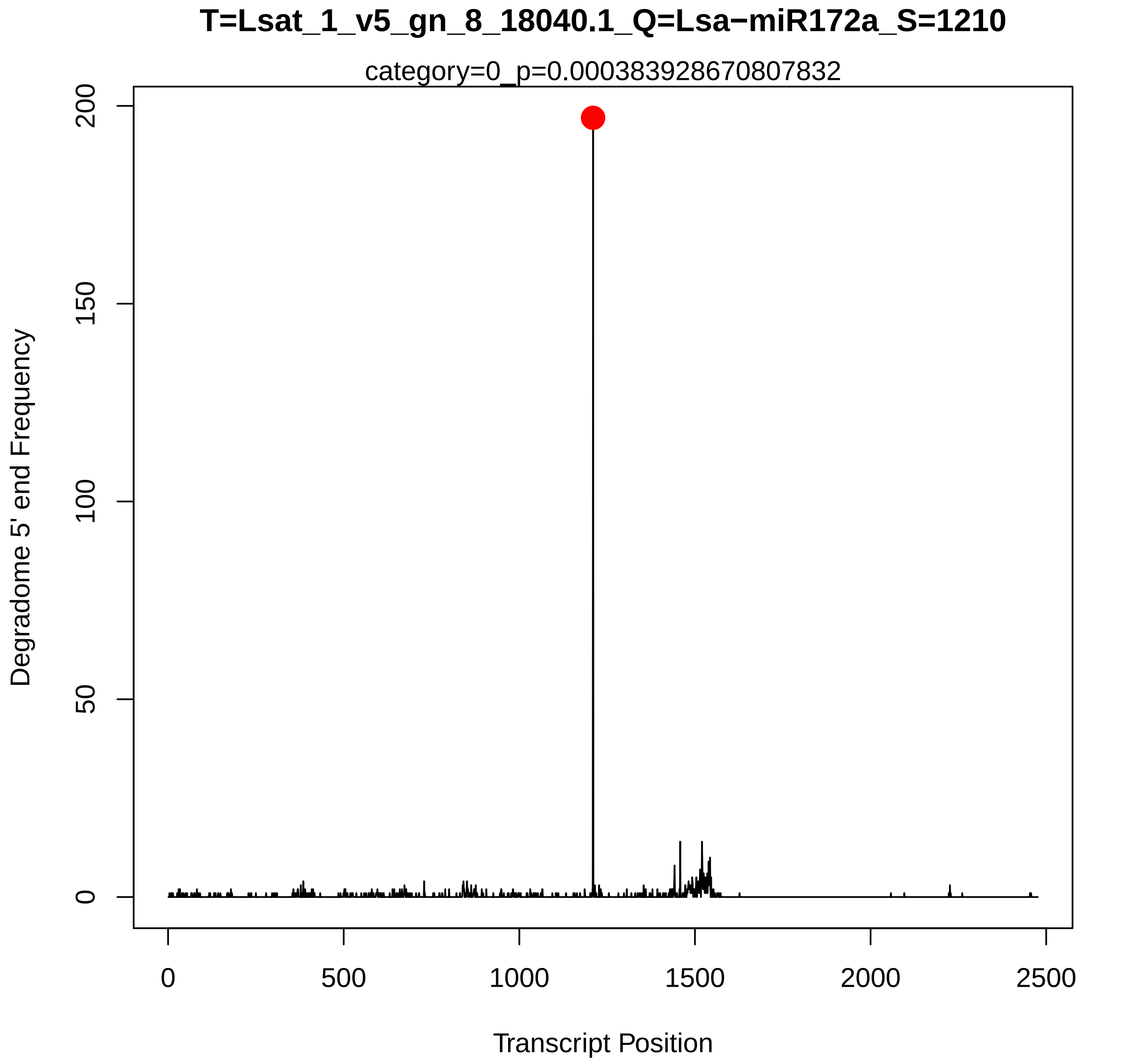
T=Lsat_1_v5_gn_8_18040.1_Q=Lsa−miR172a_S=1210
category=0_p=0.000383928670807832
Degradome 5' end Frequency
0
50
100
150
200
0
500
1000
1500
2000
2500
Transcript Position
